# Surveillance for Foodborne Disease Outbreaks — United States, 2009–2015

**DOI:** 10.15585/mmwr.ss6710a1

**Published:** 2018-07-27

**Authors:** Daniel Dewey-Mattia, Karunya Manikonda, Aron J. Hall, Matthew E. Wise, Samuel J. Crowe

**Affiliations:** 1 National Center for Emerging and Zoonotic Infectious Diseases, CDC; 2 National Center for Immunization and Respiratory Diseases, CDC

## Abstract

**Problem/Condition:**

Known foodborne disease agents are estimated to cause approximately 9.4 million illnesses each year in the United States. Although only a small subset of illnesses are associated with recognized outbreaks, data from outbreak investigations provide insight into the foods and pathogens that cause illnesses and the settings and conditions in which they occur.

**Reporting Period:**

2009–2015

**Description of System:**

The Foodborne Disease Outbreak Surveillance System (FDOSS) collects data on foodborne disease outbreaks, which are defined as the occurrence of two or more cases of a similar illness resulting from the ingestion of a common food. Since the early 1960s, foodborne outbreaks have been reported voluntarily to CDC by state, local, and territorial health departments using a standard form. Beginning in 2009, FDOSS reporting was made through the National Outbreak Reporting System, a web-based platform launched that year.

**Results:**

During 2009–2015, FDOSS received reports of 5,760 outbreaks that resulted in 100,939 illnesses, 5,699 hospitalizations, and 145 deaths. All 50 states, the District of Columbia, Puerto Rico, and CDC reported outbreaks. Among 2,953 outbreaks with a single confirmed etiology, norovirus was the most common cause of outbreaks (1,130 outbreaks [38%]) and outbreak-associated illnesses (27,623 illnesses [41%]), followed by *Salmonella* with 896 outbreaks (30%) and 23,662 illnesses (35%). Outbreaks caused by *Listeria*, *Salmonella*, and Shiga toxin-producing *Escherichia coli* (STEC) were responsible for 82% of all hospitalizations and 82% of deaths reported. Among 1,281 outbreaks in which the food reported could be classified into a single food category, fish were the most commonly implicated category (222 outbreaks [17%]), followed by dairy (136 [11%]) and chicken (123 [10%]). The food categories responsible for the most outbreak-associated illnesses were chicken (3,114 illnesses [12%]), pork (2,670 [10%]), and seeded vegetables (2,572 [10%]). Multistate outbreaks comprised only 3% of all outbreaks reported but accounted for 11% of illnesses, 34% of hospitalizations, and 54% of deaths.

**Interpretation:**

Foodborne disease outbreaks provide information about the pathogens and foods responsible for illness. Norovirus remains the leading cause of foodborne disease outbreaks, highlighting the continued need for food safety improvements targeting worker health and hygiene in food service settings. Outbreaks caused by *Listeria*, *Salmonella*, and STEC are important targets for public health intervention efforts, and improving the safety of chicken, pork, and seeded vegetables should be a priority.

**Public Health Action:**

The causes of foodborne illness should continue to be tracked and analyzed to inform disease prevention policies and initiatives. Strengthening the capacity of state and local health departments to investigate and report outbreaks will assist with these efforts through identification of the foods, etiologies, and settings linked to these outbreaks.

## Introduction

Approximately 800 foodborne disease outbreaks are reported in the United States each year, accounting for approximately 15,000 illnesses, 800 hospitalizations, and 20 deaths (*1*). Outbreak-associated foodborne illnesses are only a small subset of the estimated 9.4 million foodborne illnesses from known pathogens that occur annually in the United States (*2*). However, the food sources and exposure settings for illnesses that are not part of outbreaks can be determined only rarely. Outbreak investigations, on the other hand, often link etiologies with specific foods, allowing public health officials, regulatory agencies, and the food industry to investigate how foods become contaminated. Foodborne outbreak data also can be used to identify emerging food safety issues and to assess whether programs to prevent illnesses from particular foods are effective.

This report summarizes foodborne disease outbreaks reported in the United States in which the first illness occurred between January 1, 2009, and December 31, 2015. The report highlights a few large outbreaks as well as novel foods and food-pathogen pairs responsible for outbreaks during the reporting period.

## Methods

A foodborne disease outbreak is defined as two or more cases of a similar illness resulting from ingestion of a common food (*3*). When exposure to a contaminated food occurs in a single state, the outbreak is classified as a single-state outbreak; when exposure occurs in two or more states, the outbreak is classified as a multistate outbreak. Local, state, and territorial health departments voluntarily report foodborne outbreaks to CDC through the Foodborne Disease Outbreak Surveillance System (FDOSS) (https://www.cdc.gov/fdoss/). CDC staff also report multistate foodborne disease outbreaks to FDOSS; these outbreaks are identified by PulseNet, the national molecular subtyping network (*4*). Initially a paper-based surveillance system, FDOSS reporting became electronic in 1998. In 2009, FDOSS was incorporated into the newly created National Outbreak Reporting System, a web-based platform that also includes reports of outbreaks attributable to waterborne, person-to-person, animal contact, environmental, and indeterminate or unknown modes of transmission. 

Etiologies reported to FDOSS include bacterial, parasitic, and viral pathogens as well as chemicals and toxins. Outbreak etiologies are classified as unknown, suspected, or confirmed. Specific criteria (i.e., laboratory testing and clinical syndrome) are used to classify etiologies of outbreaks as suspected or confirmed (*5*). An outbreak is categorized as a multiple etiology outbreak if more than one agent is reported.

Foods and ingredients are identified as outbreak sources (i.e., implicated) using one or more of the following types of evidence: epidemiologic, laboratory, traceback, environmental assessment, or other data. Some outbreak investigations do not identify a source and in these instances the food is reported as unknown. CDC categorizes foods implicated in outbreak investigations on the basis of a hierarchical scheme (*6*). One of 24 food categories (e.g., mollusks) is assigned if a single contaminated ingredient (e.g., raw oysters) is reported as the source or if all implicated ingredients belong to the same category (e.g., raw oysters and raw clams). When a food or contaminated ingredient cannot be assigned to a single category, the outbreak is classified as not attributed to a single food category (*7*). The place where the implicated food was prepared is reported as one of 23 locations (e.g., a camp, farm, grocery store, or private home).

Population-based reporting rates were calculated for each state by use of U.S. Census Bureau estimates of the mid-year state populations for 2009–2015 (*8*). This report includes all foodborne outbreaks with a date of first illness onset from January, 1, 2009, through December, 31, 2015, but reported to FDOSS and finalized as of April 10, 2017.

## Results

**FIGURE 1:**
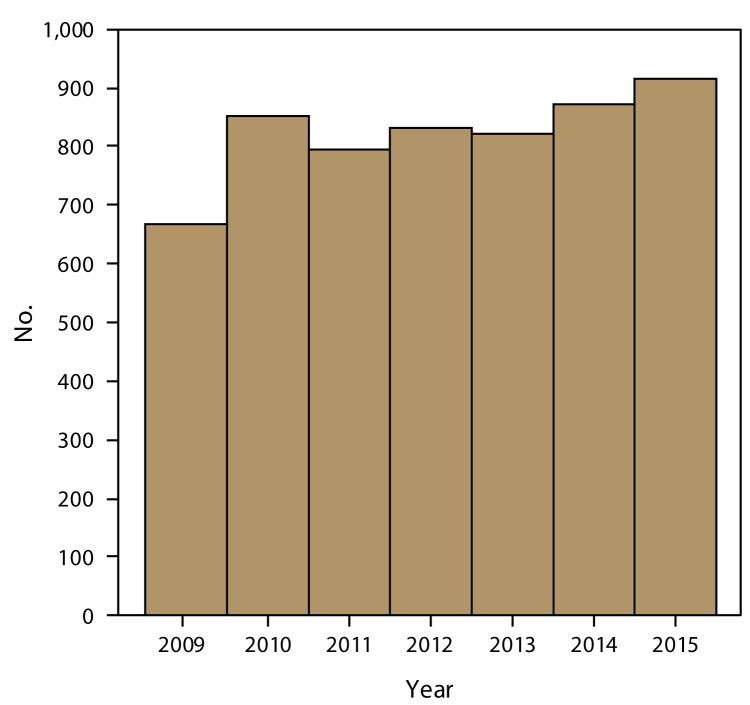
Number of foodborne disease outbreaks, by year — Foodborne Disease Outbreak Surveillance System, United States and Puerto Rico, 2009–2015

**FIGURE 2:**
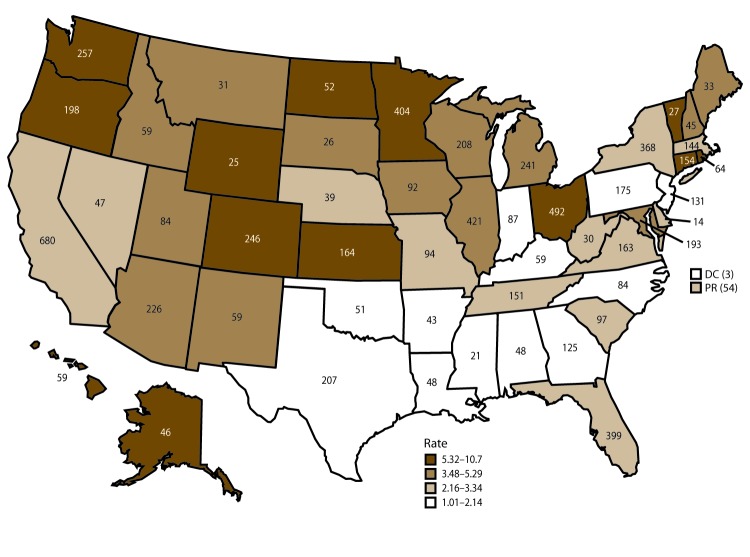
Number*and rate^†^ of reported foodborne disease outbreaks — Foodborne Disease Outbreak Surveillance System, United States and Puerto Rico 2009–2015 **Abbreviations:** DC = District of Columbia; PR = Puerto Rico. *Total number of reported outbreaks in each area (N = 5,760), includes 177 multistate outbreaks (i.e., outbreaks in which exposure occurred in more than one state) assigned as an outbreak to each state involved. Multistate outbreaks involved a median of seven states (range: 2–45). ^†^ Per 1 million population using U.S. Census Bureau estimates of the mid-year populations for 2009–2015. Source: US Census Bureau. Population and housing unit estimates. Washington, DC: US Department of Commerce, US Census Bureau; 2016. https://www.census.gov/programssurveys/popest.html. Cut points for outbreak rate categories determined by using quartiles.

During 2009–2015, FDOSS received reports of 5,760 outbreaks, resulting in 100,939 illnesses, 5,699 hospitalizations, and 145 deaths (Figure 1). Outbreaks were reported by all 50 states, the District of Columbia, Puerto Rico, and CDC (Figure 2). The single-state outbreak reporting rate was 2.6 outbreaks per 1 million population. The overall national reporting rate (which includes multistate outbreaks) during 2009–2015 was also 2.6 outbreaks per 1 million population. Single-state outbreaks accounted for 5,583 (97%) of all outbreaks with 89,907 cases (median: 8 cases per outbreak; range: 2–800 cases). Four percent of these ill persons (3,733) were reported as being hospitalized. Multistate outbreaks accounted for 177 (3%) of all outbreaks with 11,032 cases (median: 20 cases per outbreak; range: 2–1,939 cases). Eighteen percent of these ill persons (1,966) were hospitalized.

### Etiologic Agents

A single confirmed etiology was reported for 2,953 (51%) outbreaks, resulting in 67,130 illnesses, 5,114 hospitalizations, and 140 deaths (Table 1). Among 2,953 outbreaks with a single confirmed etiology, norovirus was the most common cause of outbreaks (1,130 outbreaks [38%]) and outbreak-associated illnesses (27,623 illnesses [41%]). *Salmonella* was the second most common single confirmed etiology reported, with 896 outbreaks (30%) and 23,662 illnesses (35%), followed by Shiga toxin-producing *Escherichia coli* (STEC) (191 outbreaks [6%]), *Campylobacter* (155 [5%]), *Clostridium perfringens* (108 [4%]), scombroid toxin (95 [3%]), ciguatoxin (80 [3%]), *Staphylococcus aureus* (35 [1%]), *Vibrio parahaemolyticus* (35 [1%]), and *Listeria monocytogenes* (35 [1%]). *Listeria*, *Salmonella*, and STEC were the most common causes of hospitalizations (82%) and deaths (82%) reported among persons in outbreaks with a single confirmed etiology.

**TABLE 1:** Number and percentage of foodborne disease outbreaks, outbreak-associated illnesses, and hospitalizations, by etiology (confirmed or suspected) — Foodborne Disease Outbreak Surveillance System, United States, 2009–2015

Etiology	Outbreaks				Illnesses				Hospitalizations				Deaths			
	CE*	SE	Total	%	CE	SE	Total	%	CE	SE	Total	%	CE	SE	Total	%
**Bacterial**																
*Salmonella* ^†^	896	53	949	23	23,662	510	24,172	30	3,168	39	3,207	60	29	0	29	20
*Escherichia coli*, Shiga toxin-producing (STEC)^§^	191	12	203	5	2,378	87	2,465	3	672	21	693	13	12	1	13	9
*Campylobacter* ^¶^	155	46	201	5	2,095	214	2,309	3	134	17	151	3	1	0	1	1
*Clostridium perfringens*	108	90	198	5	5,132	2,702	7,834	10	16	2	18	0	4	0	4	3
*Staphylococcus aureus*	35	40	75	2	1,255	426	1,681	2	69	17	86	2	0	0	0	0
*Bacillus cereus*	23	42	65	2	551	288	839	1	2	4	6	0	0	0	0	0
*Vibrio parahaemolyticus*	35	14	49	1	227	53	280	0	18	2	20	0	0	0	0	0
Shigella**	32	7	39	1	1,193	33	1,226	1	108	2	110	2	1	0	1	1
*Listeria monocytogenes*	35	1	36	1	380	8	388	0	334	7	341	6	74	1	75	52
*Clostridium botulinum*	19	2	21	1	85	6	91	0	72	6	78	1	4	0	4	3
*Escherichia coli*, Enterotoxigenic	6	1	7	0	437	19	456	1	1	0	1	0	0	0	0	0
*Staphylococcus* spp.	2	4	6	0	38	15	53	0	0	0	0	0	0	0	0	0
*Yersinia enterocolitica*	3	1	4	0	20	4	24	0	7	0	7	0	1	0	1	1
*Vibrio cholerae*	1	2	3	0	3	14	17	0	3	1	4	0	1	0	1	1
*Streptococcus*, Group A	2	1	3	0	72	40	112	0	0	0	0	0	0	0	0	0
*Escherichia coli*, Enteroaggregative	3	0	3	0	50	0	50	0	0	0	0	0	0	0	0	0
*Vibrio* other	2	0	2	0	7	0	7	0	3	0	3	0	0	0	0	0
*Vibrio vulnificus*	0	1	1	0	0	2	2	0	0	1	1	0	0	1	1	1
*Aeromonas hydrophila*	0	1	1	0	0	4	4	0	0	0	0	0	0	0	0	0
*Coxiella burnetti*	0	1	1	0	0	5	5	0	0	1	1	0	0	0	0	0
*Francisella novicida*	1	0	1	0	3	0	3	0	3	0	3	0	1	0	1	1
*Brucella* spp.	1	0	1	0	4	0	4	0	1	0	1	0	0	0	0	0
*Clostridium* other	1	0	1	0	12	0	12	0	0	0	0	0	0	0	0	0
*Escherichia coli*, Enteropathogenic	1	0	1	0	30	0	30	0	0	0	0	0	0	0	0	0
*Enterococcus faecalis*	1	0	1	0	13	0	13	0	0	0	0	0	0	0	0	0
Other	0	34	34	1	0	469	469	1	0	0	0	0	0	0	0	0
Subtotal	1,553	353	1,906	47	37,647	4,899	42,546	52	4,611	120	4,731	88	128	3	131	92
**Chemical and toxin**																
Scombroid toxin/histamine	95	6	101	2	280	19	299	0	1	1	2	0	0	0	0	0
Ciguatoxin	80	13	93	2	294	43	337	0	32	7	39	1	0	0	0	0
Mycotoxins	13	1	14	0	36	6	42	0	22	0	22	0	4	0	4	3
Puffer fish tetrodotoxin	3	0	3	0	9	0	9	0	4	0	4	0	0	0	0	0
Paralytic shellfish poison	3	0	3	0	12	0	12	0	6	0	6	0	0	0	0	0
Pesticides	2	0	2	0	42	0	42	0	2	0	2	0	0	0	0	0
Amnesic shellfish poison	1	0	1	0	2	0	2	0	2	0	2	0	0	0	0	0
Other	20	20	40	1	106	175	281	0	20	6	26	0	1	0	1	1
Subtotal	217	40	257	6	781	243	1,024	1	89	14	103	2	5	0	5	3
**Parasitic**																
*Cryptosporidium*	10	2	12	0	160	22	182	0	6	2	8	0	0	0	0	0
*Trichinella*	8	1	9	0	30	3	33	0	7	1	8	0	0	0	0	0
*Cyclospora*	9	0	9	0	432	0	432	1	17	0	17	0	0	0	0	0
*Giardia*	3	0	3	0	12	0	12	0	1	0	1	0	0	0	0	0
Subtotal	30	3	33	1	634	25	659	1	31	3	34	1	0	0	0	0
**Viral**																
Norovirus	1,130	740	1,870	46	27,623	9,413	37,036	45	275	99	374	7	7	0	7	5
Hepatitis A	15	0	15	0	260	0	260	0	107	0	107	2	0	0	0	0
Sapovirus	7	1	8	0	127	3	130	0	1	0	1	0	0	0	0	0
Rotavirus	1	1	2	0	58	28	86	0	0	1	1	0	0	0	0	0
Astrovirus	0	1	1	0	0	22	22	0	0	0	0	0	0	0	0	0
Other	0	2	2	0	0	25	25	0	0	0	0	0	0	0	0	0
Subtotal	1,153	745	1,898	46	28,068	9,491	37,559	46	383	100	483	9	7	0	7	5
**Single etiology^††^**	**2,953**	**1,141**	**4,094**	**71**	**67,130**	**14,658**	**81,788**	**81**	**5,114**	**237**	**5,351**	**94**	**140**	**3**	**143**	**99**
**Multiple etiologies^§§^**	**33**	**50**	**83**	**1**	**925**	**1,070**	**1,995**	**2**	**56**	**21**	**77**	**1**	**0**	**0**	**0**	**0**
**Unknown etiology^¶¶^**	**0**	**0**	**1,583**	**27**	**0**	**0**	**15,728**	**17**	**0**	**271**	**271**	**5**	**0**	**0**	**2**	**1**
**Total**	**2,986**	**1,191**	**5,760**	**100**	**68,055**	**15,728**	**100,939**	**100**	**5,170**	**258**	**5,699**	**100**	**140**	**3**	**145**	**100**

**Abbreviations:** CE = confirmed etiology; SE = suspected etiology. *Guidelines for reporting agencies are to consider an etiology confirmed if it meets confirmation criteria (https://www.cdc.gov/foodsafety/outbreaks/investigating-outbreaks/confirming_diagnosis.html); otherwise, it is considered suspected. Agents that are not listed in confirmation criteria or that are not known to cause illness are sometimes reported as confirmed or suspected etiologies.

^†^*Salmonella* serotypes causing more than five outbreaks were Enteritidis (264 outbreaks), Typhimurium (102), Newport (73), Heidelberg (49), I 4,[5],12:i:- (41), Javiana (37), Braenderup (29), Infantis (24), Montevideo (20), Muenchen (18), Thompson (17), Saintpaul (16), Oranienburg (15), Paratyphi B (10), Uganda (9), Agona (8), Typhimurium var Cope (8), Hadar (7), Mbandaka (7), Miami (6), and Virchow (6).

^§ ^STEC serogroups O157 (156 outbreaks), O26 (14), O111 (7), O121 (6), O145 (5), multiple serogroups (4), O45 (4), O103 (3), unknown serogroup (3), and O186 (1).

^¶ ^*Campylobacter jejuni* (140 outbreaks), *Campylobacter* unknown species (49), *Campylobacter* multiple species (6), *Campylobacter coli* (5), and *Campylobacter* other (1).

** *Shigella sonnei *(33 outbreaks), *Shigella flexneri* (4), and *Shigella* unknown species (2).

^†† ^The denominator for the etiology percentages is the single etiology total. The denominator for the single etiology, multiple etiologies, and unknown etiology is the total. Because of rounding, numbers might not add up to the single etiology total or the total.

^§§ ^If at least two etiologies are confirmed in an outbreak, it is considered a confirmed multiple etiology outbreak; otherwise it is considered a suspected multiple etiology outbreak.

^¶¶^ An etiologic agent was not confirmed or suspected based on clinical, laboratory, or epidemiologic information.

### Location of Food Preparation

A location of preparation was provided for 5,022 outbreak reports (87%), with 4,696 (94%) indicating a single location (Table 2**)**. Among outbreaks reporting a single location of preparation, restaurants were the most common location (2,880 outbreaks [61%]), followed by catering or banquet facilities (636 [14%]) and private homes (561 [12%]). Sit-down dining style restaurants (2,239 [48%]) were the most commonly reported type of restaurant. The locations of food preparation with the most outbreak-associated illnesses were restaurants (33,465 illnesses [43%]), catering or banquet facilities (18,141 [24%]), and institutions, such as schools (9,806 [13%]). The preparation location with the largest average number of illnesses per outbreak was institutions (46.5), whereas restaurants had the smallest (11.6).

**TABLE 2:** Number and percentage of foodborne disease outbreaks and outbreak-associated illnesses, by location of food preparation — Foodborne Disease Outbreak Surveillance System, United States, 2009–2015

Location	Outbreaks		Illnesses		Mean illnesses per outbreak
	No.	%	No.	%	
**Restaurant**	**2,880**	**61**	**33,465**	**43**	**12**
Sit-down dining	2,239	48	25,150	33	11
Fast-food	369	8	4,414	6	12
Buffet	9	0	97	0	11
Other or unknown type	229	5	3,231	4	14
Multiple types	34	1	573	1	17
**Catering or banquet facility**	**636**	**14**	**18,141**	**24**	**29**
**Private home**	**561**	**12**	**8,080**	**10**	**14**
**Institutional location**	**211**	**4**	**9,806**	**13**	**46**
School	69	1	2,164	3	31
Prison or jail	67	1	5,077	7	76
Camp	29	1	904	1	31
Day care	7	0	193	0	28
Office or indoor workplace	26	1	937	1	36
Other	13	0	531	1	41
**Other location**	**26**	**1**	**482**	**1**	**19**
**Other commercial location**	**258**	**5**	**4,284**	**6**	**17**
Grocery store	104	2	1,611	2	15
Fair, festival, or temporary mobile service	37	1	620	1	17
Farm or dairy	79	2	1,178	2	15
Other	38	1	875	1	23
**Hospital or nursing home**	**68**	**1**	**1,527**	**2**	22
Nursing home	55	1	1,349	2	25
Hospital	13	0	178	0	14
**Other private location**	**44**	**1**	**1,203**	**2**	**27**
Place of worship	32	1	1,014	1	32
Picnic	5	0	37	0	7
Other	7	0	152	0	22
**Hotel or motel**	**8**	**0**	**151**	**0**	**19**
**Ship or boat**	**4**	**0**	**31**	**0**	**8**
Single location*	**4,696**	**82**	**77,170**	**76**	**16**
**Multiple locations**	**326**	**6**	**10,920**	**11**	**33**
**Unknown location**	**738**	**13**	**12,849**	**13**	**17**
**Total**	**5,760**	**100**	**100,939**	**100**	**18**

* The denominator for the location percentages is the single location total. The denominator for the single location, multiple locations, and unknown location is the total. Numbers might not add up to the single location total or the total due to rounding.

### Foods

Outbreak investigators identified a food in 2,442 outbreaks (42%). These outbreaks resulted in 51,341 illnesses (51%) (Table 3). The food reported belonged to a single food category in 1,281 outbreaks (22%). The food category most commonly implicated was fish (222 outbreaks [17%]), followed by dairy (136 [11%]) and chicken (123 [10%]). The food categories responsible for the most outbreak-associated illnesses were chicken (3,114 illnesses [12%]), pork (2,670 [10%]), and seeded vegetables (2,572 [10%]). Scombroid toxin in fish was the single confirmed etiology and food category pair responsible for the most outbreaks (85), followed by ciguatoxin in fish (72) and *Campylobacter* in dairy (60) (Table 4). The pathogen-food category pairs that caused the most outbreak-associated illnesses were *Salmonella* in eggs (2,422 illnesses), *Salmonella* in seeded vegetables (2,203), and *Salmonella* in chicken (1,941). In comparison, scombroid toxin and ciguatoxin outbreaks from fish resulted in 519 outbreak-associated illnesses, an average of three illnesses per outbreak. Outbreaks of *Salmonella* infections from seeded vegetables resulted in an average of 88 illnesses per outbreak, and outbreaks of *Salmonella* infections from eggs resulted in an average of 78 illnesses per outbreak.

**TABLE 3:** Number and percentage of foodborne disease outbreaks and outbreak-associated illnesses, by food category — Foodborne Disease Outbreak Surveillance System, United States and Puerto Rico, 2009–2015

Food category*	Outbreaks		Illnesses	
	No.	%	No.	%
**Aquatic animal**				
Crustaceans	12	1	74	0
Mollusks^†^	105	8	846	3
Fish	222	17	1,353	5
Other aquatic animals	5	0	15	0
Subtotal	344	27	2,288	9
**Land animal**				
Dairy^§^	136	11	1,639	6
Eggs	36	3	2,470	9
Beef	106	8	1,934	7
Pork	89	7	2,670	10
Other meat (e.g., sheep or goat)	6	0	50	0
Chicken	123	10	3,114	12
Turkey	50	4	1,675	6
Other poultry	6	0	71	0
Game	13	1	86	0
Subtotal	565	44	13,709	52
**Plant**				
Oils and sugars	4	0	18	0
Fungi	16	1	56	0
Sprouts	21	2	766	3
Root and other underground vegetables^¶^	20	2	383	1
Seeded vegetables**	44	3	2,572	10
Herbs	7	1	476	2
Vegetable row crops^††^	81	6	1,972	7
Fruits^§§^	78	6	2,420	9
Grains and beans^¶¶^	52	4	838	3
Nuts and seeds***	11	1	245	1
Subtotal	334	26	9,746	37
**Other**	**38**	**3**	**807**	**3**
**Food reported, attributed to a single food category^†††^**	**1,281**	**22**	**26,550**	**26**
**Food reported, not attributed to a single food category**	**1,161**	**20**	**24,791**	**25**
**No food reported**	**3,318**	**58**	**49,598**	**49**
**Total^†††^**	**5,760**	**100**	**100,939**	**100**

* **Source:** Interagency Food Safety Analytics Collaboration (IFSAC) food categorization scheme (https://www.cdc.gov/foodsafety/ifsac/projects/food-categorization-scheme.html).

^† ^Bivalve mollusks (102 outbreaks) and nonbivalve mollusks (3).

^§ ^Unpasteurized dairy products (109 outbreaks), pasteurized dairy products (20), and pasteurization unknown (7).

^¶ ^Tubers (12 outbreaks), roots (5), and bulbs (3).

** Solanaceous seeded vegetables (23 outbreaks), vine-grown seeded vegetables (11), legumes (7), other seeded vegetables (2), and seeded vegetables not further classified (1).

^†† ^Leafy vegetables (77 outbreaks) and stem vegetables (4).

^§§ ^Fruits not further classified (24 outbreaks), pome fruits (15), melons (14), small fruits (11), sub-tropical fruits (7), tropical fruits (5), and stone fruits (2).

^¶¶ ^Grains (32 outbreaks), beans (15), and grains and beans not further classified (5).

*** Nuts (8 outbreaks) and seeds (3).

**^††† ^**The denominator for the food category percentages is the "food reported, attributed to a single food category" total. The total comprises "food reported attributed to a single food category," "food reported, not attributed to a single food category," and "no food reported." Numbers might not add up exactly due to rounding.

**TABLE 4:** Most common confirmed pathogen-food category pairs resulting in outbreaks, outbreak-associated illnesses, hospitalizations, and deaths — Foodborne Disease Outbreak Surveillance System, United States and Puerto Rico, 2009–2015

Characteristic	Food category*	No. outbreaks	No. illnesses	No. hospitalizations	No. deaths
**Top 5 pathogen-food category pairs resulting in outbreaks**					
**Etiology**					
Scombroid toxin/histamine	Fish	85	250	1	0
Ciguatoxin	Fish	72	269	31	0
*Campylobacter*	Dairy	60	917	51	1
*Salmonella*	Chicken	49	1,941	372	0
*Salmonella*	Pork	43	1,539	206	3
**Top 5 pathogen-food category pairs resulting in outbreak-associated illnesses**					
**Etiology**					
*Salmonella*	Eggs	31	2,422	41	1
*Salmonella*	Seeded vegetables	25	2,203	419	7
*Salmonella*	Chicken	49	1,941	372	0
*Salmonella*	Pork	43	1,539	206	3
*Campylobacter*	Dairy	60	917	51	1
**Top 5 pathogen-food category pairs resulting in outbreak-associated hospitalizations**					
**Etiology**					
*Salmonella*	Seeded vegetables	25	2,203	419	7
*Salmonella*	Chicken	49	1,941	372	0
*Salmonella*	Fruits	24	838	227	6
*Salmonella*	Pork	43	1,539	206	3
*Listeria monocytogenes*	Fruits	3	184	179	41
**Top 5 pathogen-food category pairs resulting in outbreak-associated deaths **					
**Etiology**					
*Listeria monocytogenes*	Fruits	3	184	179	41
*Listeria monocytogenes*	Dairy	14	106	70	14
*Salmonella*	Seeded vegetables	25	2,203	419	7
*Salmonella*	Fruits	24	838	227	6
*Listeria monocytogenes*	Vegetable row crops	2	29	29	6

*Source: Interagency Food Safety Analytics Collaboration (IFSAC) food categorization scheme: https://www.cdc.gov/foodsafety/ifsac/projects/food-categorization-scheme.html.

Several novel food vehicles caused outbreaks during the study period. In 2011, an outbreak of *Salmonella* serotype Enteritidis infections linked to pine nuts imported from Turkey resulted in 53 illnesses and two hospitalizations. In 2014, an outbreak of *Salmonella* serotypes Gaminara, Hartford, and Oranienburg in chia seed powder imported from Canada caused 45 illnesses and seven hospitalizations. An outbreak of STEC serogroups O26 and O121 infections that began in 2015 was linked to raw wheat flour produced in the United States; it resulted in 56 illnesses and 16 hospitalizations in 24 states. An outbreak of *Salmonella* serotype Virchow infections attributable to moringa leaf powder imported from South Africa began in 2015 and caused 35 illnesses and six hospitalizations in 24 states. It was an ingredient of an organic powdered shake mix branded to be used as a meal replacement.

### Multistate Outbreaks

Multistate outbreaks comprised only 3% of outbreaks but were responsible for 11% of illnesses, 34% of hospitalizations, and 54% of deaths. Multistate outbreaks involved a median of seven states with a range of two to 45 states in which exposure occurred. The largest of the 177 multistate outbreaks was caused by *Salmonella* serotype Enteritidis and due to contaminated shell eggs. An estimated 1,939 persons were infected in 10 states beginning in 2010. An outbreak of *Salmonella* serotype Poona infections attributed to cucumbers in 2015 had the second highest number of illnesses (907 illnesses in 40 states). This outbreak also had the most outbreak-associated hospitalizations (204 [22% of cases]). An outbreak of *Salmonella* serotype Heidelberg infections attributed to chicken during 2013–2014 had the second most hospitalizations (200 [32% of cases]) and involved persons from 29 states and Puerto Rico. An outbreak of *Listeria monocytogenes* infections attributed to cantaloupes in 28 states in 2011 had the most deaths (33 [22% of cases]), followed in 2014 by an outbreak in 12 states of *Listeria monocytogenes* infections attributed to caramel apples, another novel food vehicle (*9*), in which seven persons (20% of cases) died.

## Discussion

Despite considerable advances in food safety in the United States during recent decades, foodborne disease outbreaks remain a serious public health problem. The majority of the outbreaks reported had relatively small case counts, and affected persons often were exposed in a single state. However, outbreaks with the largest case counts and most severe outcomes (e.g. highest proportion of ill persons hospitalized and most deaths) typically involved exposures in multiple states, reflecting factors such as the geographical distribution of the implicated food and the characteristics of the pathogens involved. Foods produced in other countries sometimes were implicated, highlighting the interconnectedness of the U.S. food supply with that of other nations, and the continued need to ensure that all foods are safe to eat (*10*).

As reported in previous summaries (*11*), norovirus remains the leading cause of foodborne disease outbreaks and outbreak-associated illnesses in the United States. Most foodborne norovirus outbreaks are associated with ready-to-eat foods contaminated during preparation by infected food workers in restaurants and other food service settings (*12*). As such, continued efforts are needed to strengthen and ensure compliance with requirements in the FDA Model Food Code (*13*), specifically those that exclude symptomatic and post-symptomatic workers, prohibit bare-hand contact with ready-to-eat foods, and ensure appropriate hand washing. Contaminated raw food products, specifically leafy vegetables, fruits, and mollusks, also have been implicated in norovirus outbreaks (*12*); thus, upstream contamination during production also should be considered in foodborne norovirus outbreak investigations.

Fish was the most frequently implicated food, but the number of illnesses associated with these outbreaks tended to be small compared with other food vehicles, largely because of the pathogens involved. Differences in outbreak size are in part attributable to how pathogens contaminate foods: toxins are produced in individual fish, whereas *Salmonella* and other bacterial pathogens, such as STEC, can contaminate large amounts of product across vast distribution chains (*14*). This helps explain why bacterial pathogens are the most common causes of multistate outbreaks and why many persons can become ill during a single bacterial disease outbreak.

Identification of novel food sources provides insight into evolving food preferences in the United States and the types of foods that pathogens can contaminate. It also raises important scientific questions regarding how these pathogens remain viable in these foods long enough to cause infection. During the study period, a few novel food vehicles were identified as the sources of multistate outbreaks of *Listeria*, *Salmonella*, and STEC infections. Some of these (chia seed powder, raw wheat flour, and moringa leaf powder) are dried, shelf-stable foods not usually considered as possible sources of illness. These outbreak reports provide additional evidence that *Salmonella* and STEC can survive extensive processing steps as well as months in a desiccated state. This ability of pathogens to remain viable combined with the long shelf life of these products emphasizes the need for clear, well-publicized product recall notices.

*Salmonella* and STEC were two of the most common causes of large outbreaks. Regulatory-focused public health interventions, such as the 2009 Egg Safety Rule, the 2011 Food Safety Modernization Act, and the 2013 *Salmonella* Action Plan, were designed and implemented in part to help ensure the safety of foods that can be contaminated by these pathogens (*15*–*17*). Some members of the food industry also are promoting a culture of food safety by requiring growers, producers, and distributors to adhere to strict safety guidelines designed to prevent contamination. Additional efforts will likely be needed by both government and industry to help control these pathogens.

## Limitations

The findings of this report are subject to at least four limitations. First, because CDC’s foodborne outbreak surveillance is dynamic and agencies can submit, update, or delete reports at any time, the results of this analysis might differ slightly from previous or future reports. Second, not all outbreaks are identified and the majority of foodborne illnesses occur outside the context of a recognized outbreak. The degree to which the food vehicles, etiologies, and locations implicated in outbreaks represent the vehicles, etiologies, and locations of sporadic foodborne illness is unknown. Third, some outbreaks have an unknown food vehicle, an unknown etiology, or both, and analyses and conclusions drawn from outbreaks with an identified food vehicle and confirmed etiology might not be representative of all outbreaks. Finally, pathogens that are not known to cause illness sometimes are reported as a confirmed or suspected etiology.

## Conclusion

Foodborne disease outbreaks remain an important public health issue. Data collected during outbreak investigations provide insight into the foods and pathogens that cause illnesses and the settings and conditions in which they occur. Continued efforts must be made to track and to analyze the causes of foodborne illness to inform targeted prevention efforts. In particular, strengthening the capacity of state and local health departments to investigate and to report outbreaks will improve foodborne disease outbreak surveillance and could help decrease the burden of foodborne illness through identification of foods, etiologies, outbreak settings, and specific points of contamination, which can inform intervention efforts.
